# Climate Anomalies and Spillover of Bat-Borne Viral Diseases in the Asia–Pacific Region and the Arabian Peninsula

**DOI:** 10.3390/v14051100

**Published:** 2022-05-20

**Authors:** Alice Latinne, Serge Morand

**Affiliations:** 1 Wildlife Conservation Society, Viet Nam Country Program, Ha Noi 100000, Vietnam; 2 Wildlife Conservation Society, Global Conservation Program, Bronx, NY 10460, USA; 3 MIVEGEC, CNRS—IRD—Montpellier Université, 911 Avenue Agropolis, BP 6450, 34394 Montpellier, France; serge.morand@umontpellier.fr; 4 Faculty of Veterinary Technology, University of Kasetsart, Bangkok 10900, Thailand; 5 Faculty of Tropical Medicine, University of Mahidol, Bangkok 10400, Thailand

**Keywords:** bat-borne virus, spillover, SARS-CoV-2, Nipah virus, Hendra virus, climate change, El Niño Southern Oscillation, event coincidence analysis, temporal analysis, structural equation modelling

## Abstract

Climate variability and anomalies are known drivers of the emergence and outbreaks of infectious diseases. In this study, we investigated the potential association between climate factors and anomalies, including El Niño Southern Oscillation (ENSO) and land surface temperature anomalies, as well as the emergence and spillover events of bat-borne viral diseases in humans and livestock in the Asia–Pacific region and the Arabian Peninsula. Our findings from time series analyses, logistic regression models, and structural equation modelling revealed that the spillover patterns of the Nipah virus in Bangladesh and the Hendra virus in Australia were differently impacted by climate variability and with different time lags. We also used event coincidence analysis to show that the emergence events of most bat-borne viral diseases in the Asia–Pacific region and the Arabian Peninsula were statistically associated with ENSO climate anomalies. Spillover patterns of the Nipah virus in Bangladesh and the Hendra virus in Australia were also significantly associated with these events, although the pattern and co-influence of other climate factors differed. Our results suggest that climate factors and anomalies may create opportunities for virus spillover from bats to livestock and humans. Ongoing climate change and the future intensification of El Niño events will therefore potentially increase the emergence and spillover of bat-borne viral diseases in the Asia–Pacific region and the Arabian Peninsula.

## 1. Introduction

Most zoonotic and vector-borne diseases are climate-sensitive, particularly to temperature or precipitations [1], and several are also sensitive to climate variability and anomalies [2,3,4]. The El Niño Southern Oscillation (ENSO), with its alternating warming (El Niño), cooling (La Niña), and neutral phases, is one of the most important climate phenomena due to its ability to modify the global atmospheric circulation and the temperature and precipitation patterns across the globe [5]. The ENSO has significant cascade effects on ecosystems [6,7,8] and agriculture productivity [9]. ENSO-related climate variability is also a known driver of the emergence and outbreaks of infectious diseases [3,10]. Outbreaks of numerous infectious diseases, such as cholera [11], Rift Valley fever [12], visceral leishmaniasis [13], dengue [14,15], Zika virus [16], and malaria [17], among others, have been linked to the ENSO. The emergences of some viral diseases of bat origin, such as the Hendra virus (HeV) in Australia and the Nipah virus (NiV) in Malaysia, have also been associated with El Niño events [18,19]. Several mechanisms by which the ENSO affects and facilitates the transmission of zoonotic diseases have been suggested, including modifications of seasonal cycles, population dynamics, and distribution ranges of vectors and hosts of zoonotic pathogens, as well as alterations of the replication and transmission patterns of these pathogens [1,3,4].

In this study, we reviewed the emergence events and recurring spillover events of bat-borne viral diseases in humans and livestock in the Asia–Pacific region and the Arabian Peninsula, i.e., two regions highly affected by El Niño/La Niña events [20]. Furthermore, we investigated the potential association between climate anomalies, El Niño/La Niña events, and these emergence and spillover events. First, we tested the potential association between the spillover events of HeV in Australia and NiV in Bangladesh, i.e., two bat-borne viruses characterized by a high number of recurring spillover events in these two regions, and climate factors (temperature, rainfall) and anomalies (ENSO and land surface temperature anomalies) using time-series analyses, logistic regression models, and structural equation modelling. Second, we assessed potential simultaneities between the emergence events of bat-borne viruses in human and livestock populations and El Niño/La Niña events using event coincidence analysis (ECA) [21]. We then discussed the potential ecological mechanisms that may explain the emergence and spillover events of bat-borne viruses in relation to climate factors and events of El Niño/La Niña.

## 2. Materials and Methods

### 2.1. Data

Bat-borne viral pathogens were defined as viruses with bats (Chiroptera) as their natural animal hosts, i.e., the long-term ecological niche of a viral population [22], or viruses whose closest viral relatives have bats as their natural hosts. The zoonotic sources of these viruses in human populations was either bats or another host species involved as an intermediate host in their emergence. Emergence events were defined as the first detection of a bat-borne viral pathogen in human or in livestock populations or the first detection of a bat-borne viral pathogen in a region significantly distant from any other regions where it was previously observed (e.g., Nipah virus emergence events in Malaysia, India, and the Philippines). Recurring spillover events correspond to subsequent pathogen detections after its first emergence in the same region. Data on the emergence of bat-borne viral diseases in human and livestock populations in the study area during the period 1990–2020 were gathered from original sources (Table 1) and from several databases (Emerging Infectious Diseases Repository (EIDR), https://eidr.ecohealthalliance.org/ (accessed on 17 May 2022); World Animal Health Information System (OIE-WAHIS), https://wahis.oie.int/#/home (accessed on 17 May 2022); PROMED). Data on recurring spillover events of the Nipah virus in Bangladesh and India (Table 2) were obtained from three studies [23,24,25], while data on recurring spillover events of the Hendra virus in Australia were obtained from the Queensland Government database (https://www.business.qld.gov.au/industries/service-industries-professionals/service-industries/veterinary-surgeons/guidelines-hendra/incident-summary (accessed on 17 May 2022)) (Table 3).

Data on ENSO values were retrieved from the National Oceanic and Atmospheric Administration (NOAA, https://www.noaa.gov, accessed on 17 May 2022). The ‘NINO 3.4’ index is the most commonly used index used to define El Niño and La Niña events and to study climate–rainfall or climate–disease connections [3]. The NINO 3.4 index is based on a 5-month running mean of the sea surface temperature (SST) in the region bounded by 5° N to 5° S, from 170° W to 120° W. El Niño (warm phase) and La Niña (cool phase) are defined when anomalies in the NINO 3.4 index exceeds +0.4 °C or −0.4 °C, respectively (NOAA, https://www.noaa.gov, accessed on 17 May 2022). The R package rsoi [34] was used to import the NINO 3.4 index values for the period 1990–2020 and the corresponding defined El Niño (warm phase) and La Niña (cool phase) phases from the NOAA website.

Data on the average monthly temperature and rainfall data in Australia and Bangladesh were also gathered from the World Bank database (https://climateknowledgeportal.worldbank.org, accessed on 17 May 2022) and data on the global land surface temperature anomalies were obtained from the NOAA (https://www.ncei.noaa.gov/access/monitoring/global-temperature-anomalies, accessed on 17 May 2022) to investigate the impact of other climate factors (rainfall, temperature) and climate variability (global land surface temperature anomalies), in addition to the ENSO anomalies, on the occurrence of recurring spillover events of HeV and NiV viruses.

### 2.2. Statistical Analyses

First, we investigated the potential association between the spillover events of HeV in Australia and NiV in Bangladesh and several climate factors. For this, we used: (1) time-series analyses to investigate the temporal association between HeV/NiV spillover events, and temperature, rainfall, ENSO, and land surface temperature anomalies and to estimate their time lag values; (2) logistic regression models to determine the significant factors (temperature, rainfall, ENSO, land surface temperature anomalies) explaining the spillover events using the time lag values computed from the results of the time-series analyses; and (3) structural equation modelling to test a causal chain of correlation that may explain the spillover events using the results of the logistic regression analyses. Second, we assessed potential simultaneities between the emergence events of all bat-borne viruses in human and livestock populations and El Niño/La Niña events using event coincidence analysis to test the hypothesis that HeV/NiV spillover events were statistically preceded by an event of El Niño/La Niña.

#### 2.2.1. Time-Series Analysis

Time-series analyses were used to study the temporal patterns of ENSO anomalies (using the NINO 3.4 index), as well as the average monthly temperature and rainfall in Australia and Bangladesh using the ncf function implemented in R [35]. The time series included 330 months in total from January 1993, i.e., one year before the first spillover event recorded in our dataset, to June 2020. The residual autocorrelation function (ACF) was examined to determine the general form of the model to be fitted. A wavelet analysis was used to decompose a time series to reveal periodic signals at each time point in the series. The wavelet analysis coefficients show the correlation magnitudes of ENSO anomalies (NINO 3.4 index), temperature, or rainfall for each year and period length of the time series (i.e., 1993 to 2020), displayed using a power spectrum over the full time series using the biwavelet and WaveletComp packages [36,37] implemented in R [38]. The ccf function was then used to compute the cross-correlation or cross-covariance between univariate series, i.e., ENSO (NINO 3.4 index); the average monthly temperature; the average monthly rainfall; land surface temperature anomalies; and either HeV or NiV recurring spillover events in Australia and in Bangladesh, respectively.

#### 2.2.2. Logistic Regression with Time Lag Analysis

Logistic regression modelling with a logit function and lag was used to test the significant effects of monthly rainfall; monthly temperature; anomalies in land surface temperature; and ENSO anomalies (NINO 3.4 index) on the recurring spillover events of HeV and NiV in Australia and Bangladesh, respectively. The most significant lag values computed by time-series cross-correlation analysis, as described above, and the glm function implemented in R with the family binomial [38] were used.

The initial general linear model with logit function was of the form:

Recurrent spillover of HeV/NiV ~ lag(NINO 3.4 index, lag value) 

+ lag(average temperature Australia/Bangladesh, lag value)

+ lag(average rainfall Australia/Bangladesh, lag value)

+ lag(global land surface temperature anomalies, lag value)

Initial models included variables with significant lag values obtained from cross-correlation time-series analysis. Final models were selected using backward selection and AIC criterion using the stepAIC function of the MASS package [39] implemented in R.

#### 2.2.3. Structural Equation Modelling

Structural equation modelling (SEM) was used to investigate the temporal relationships between recurring outbreaks of HeV and NiV, respectively, in Australia and Bangladesh, in relation to monthly rainfall, monthly temperature, anomalies in land surface temperature, and the NINO 3.4 index values. SEM combines measurement models (e.g., reliability) with structural models (e.g., regression), thus testing a chain of causality (path analysis) between outbreaks of HeV and NiV in Australia and Bangladesh and these climatic factors. SEM was performed using the ‘piecewiseSEM’ package [40]. The following structural equation model was tested for the period from January 1993 to June 2020 of the dataset:

F (outbreaks of HeV or NiV) = f1 (lag NINO 3.4 index) + f2 (lag temperature Australia or Bangladesh) + f2 (lag rainfall Australia or Bangladesh) + f2 (lag global land surface temperature anomalies) + b1

G (lag temperature Australia or Bangladesh) = g1 (lag NINO 3.4 index) + b2

H (lag rainfall Australia or Bangladesh) = h1 (lag NINO 3.4 index) + b3

I (lag anomalies of the land surface temperature) = i (lag NINO 3.4 index) + b4

with lag values computed by time-series cross-correlation analysis.

#### 2.2.4. Event Coincidence Analysis

Finally, event coincidence analysis (ECA) was used to test if events of a given type are causally influenced by the timing of events of second type [41] and to investigate the statistical interdependence between emergence and spillover events of bat-borne viruses and El Niño/La Niña events. ECA was implemented in the CoinCalc R package [42] to test whether the observed coincidence rates are significantly different from two independent random events [41]. ECA defines the precursor coincidence rate (pcr) and the trigger coincidence rate (tcr). The pcr describes the fraction of first-type events, i.e., emergence/spillover events, preceded by at least one second-type event, i.e., El Niño/La Niña events. The tcr describes the fraction of second-type events, i.e., El Niño/La Niña events, followed by at least one first-type event, i.e., emergence/spillover events (see [41]). CoinCalc computed the probability of the precursor and trigger coincidence rates occurring by chance, with the null hypothesis that the observed precursor and trigger coincidence rates can be explained by two independent series of randomly distributed events [42]. The *p*-value of the corresponding analytical significance test corresponds to the probability that the two types of events are randomly distributed and independent of each other (following two independent Poisson processes) and sufficiently rare.

We tested the hypothesis that the pcr describing the emergence of bat-borne viruses or recurrent spillover events of NiV and HeV were statistically preceded by an El Niño/La Niña event, while the tcr did not depart from a random association. For the analysis of the recurrent spillover events of NiV in Bangladesh and HeV in Australia, the time lag values estimated in months using cross-correlation among the time series of outbreak events and NINO 3.4 index values were used. The time lag values were also moved around their estimates to explore the stability and constancy of the association given by ECA.

## 3. Results

**Figure 1:**
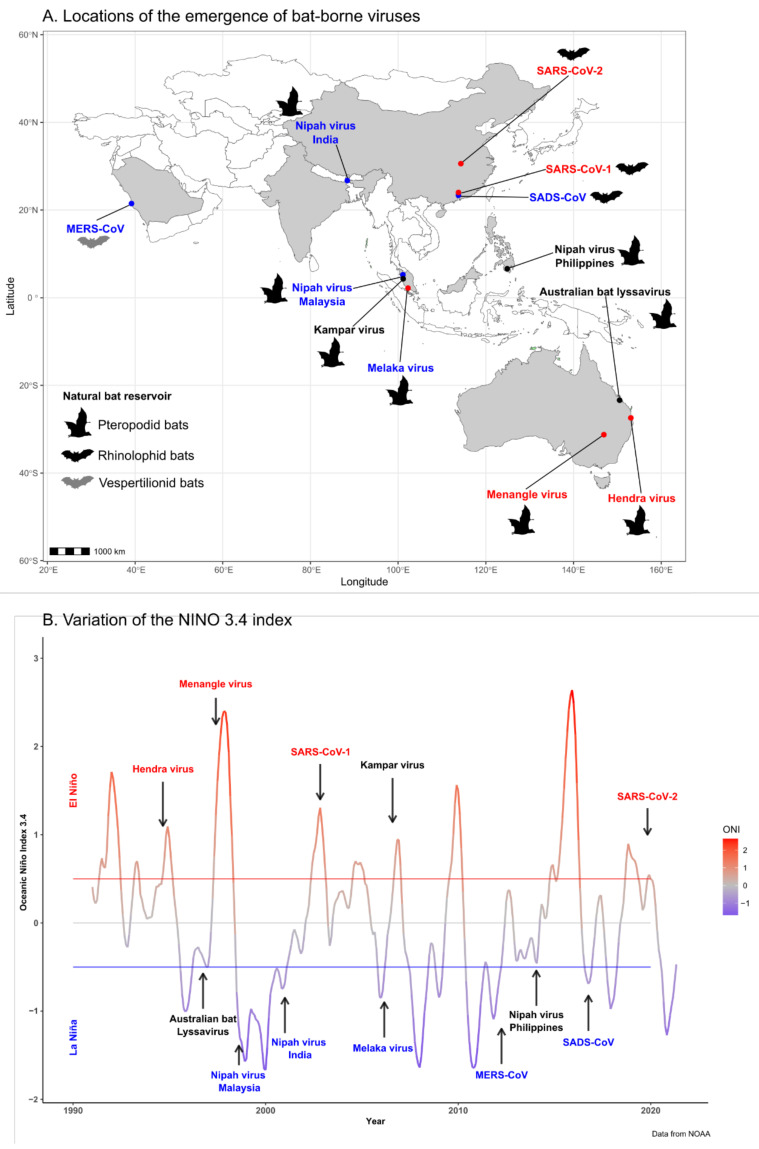
(**A**) Map showing the locations of emergence of bat-borne viruses in the Asia–Pacific region and the Arabian Peninsula (see Table 1) and the bat reservoir of each virus. Virus names are colored according to the ENSO phase at the time of their emergence: neutral phase (black), cool-phase La Niña (blue), or warm-phase El Niño (red). (**B**) Variations of the NINO 3.4 index characterizing the El Niño Southern Oscillation (ENSO) retrieved from the National Oceanic and Atmospheric Administration (NOAA, https://www.noaa.gov, accessed on 17 May 2022) from 1990 to 2020. Red and blue threshold lines indicate warming El Niño or cooling La Niña climate anomalies, respectively. Arrows indicate the emergence time of new bat-borne viruses in the Asia–Pacific region and the Arabian Peninsula (see Table 1). Virus names are colored according to the ENSO phase at the time of their emergence: neutral phase (black), cool-phase La Niña (blue), or warm-phase El Niño (red).

A total of ten bat-borne viruses, belonging to the Coronaviridae (*n* = 4), Paramyxoviridae (*n* = 3), Reoviridae (*n* = 2), and Rhabdoviridae (*n* = 1) families, emerged in the Asia–Pacific region and the Arabian Peninsula in the period 1990–2020 (Table 1; Figure 1). Nine of these viruses emerged in humans, while the swine acute diarrhea syndrome coronavirus (SADS-CoV) emerged in swine populations but was never detected in humans. Natural bat hosts of the Coronaviridae viruses were vespertilionid and rhinolophid bats, while their intermediate hosts included several mammal species. Pteropodid bats were the hosts of the emerging Paramyxoviridae, Reoviridae, and Rhabdoviridae, and livestock (horses and pigs) were involved as intermediate hosts in the emergence of HeV and NiV. Most of these viruses emerged in a single geographic area; only NiV emerged in several distant locations over a 16-year period. It first emerged in Malaysia in 1998, before then emerging in Bangladesh and India in 2001 and in the Philippines in 2014 (Table 1; Figure 1). After their first emergence, NiV and HeV then regularly spilled over in Bangladesh and Australia, respectively (Table 2 and Table 3). 

Five emergence events, including NiV in Malaysia and India, Melaka virus, Middle East respiratory syndrome coronavirus (MERS-CoV), and SADS-CoV, occurred during a cool phase (La Niña event), while four of them, i.e., HeV, Menangle virus, and severe acute respiratory syndrome coronavirus 1 and 2 (SARS-CoV-1 and -2), occurred during a warm phase (El Niño event) (Figure 1; Table 1). The remaining three emergence events of bat-borne viruses, Kampar virus, Australian bat lyssavirus, and NiV in the Philippines occurred during a neutral phase. However, the spillover of the Australian bat lyssavirus to a human might not be considered as a natural emergence since the infection was acquired from a pet fruit bat living in captivity [26]. It should also be noted that the emergence of NiV in the Philippines in March 2014 followed the major volcanic activity of Mayon volcano that started in May 2013 [43].

### 3.1. Time-Series Analyses for HeV and NiV

**Figure 2:**
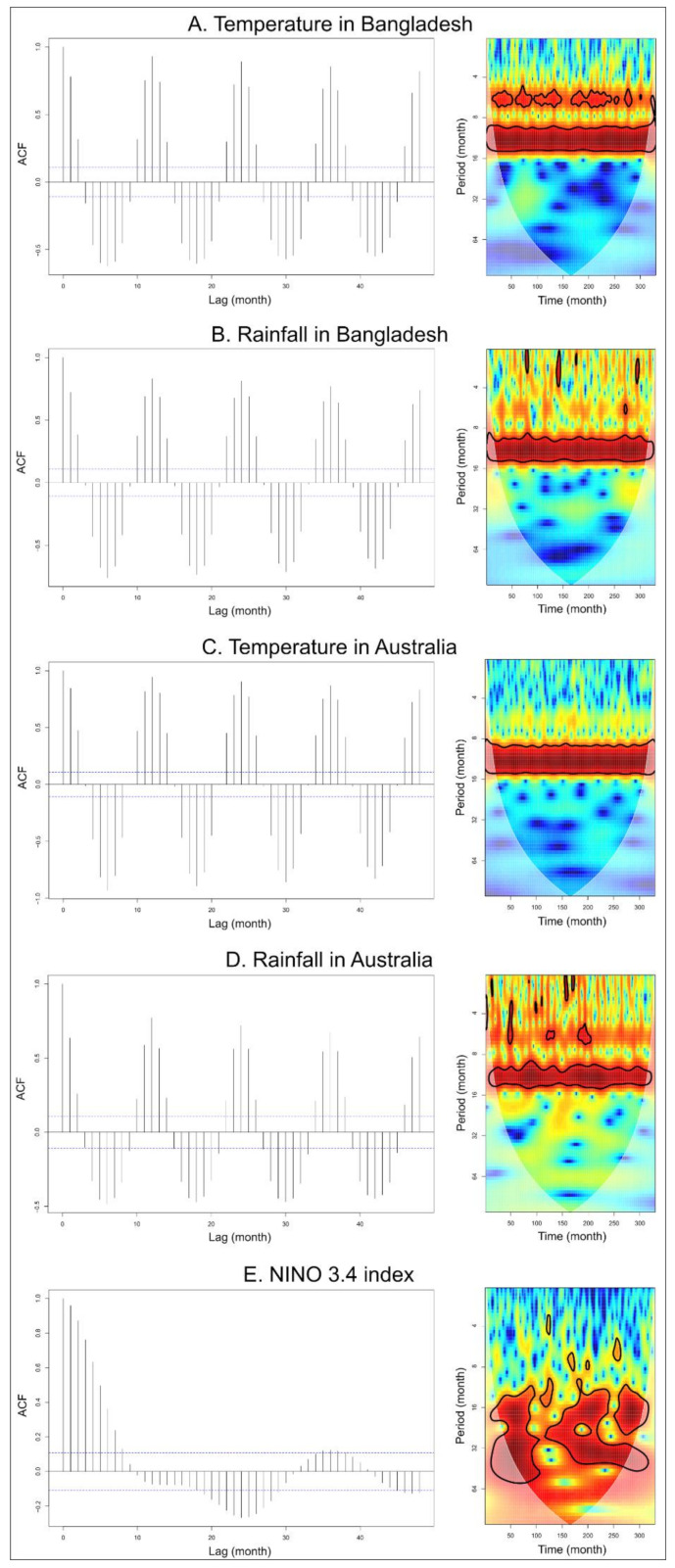
Time series and residual autocorrelation function (ACF) with significant auto-correlation values in dashed lines (left column) and wavelet power spectrum (right column) from January 1993 to June 2020 (330 months) of (**A**) monthly temperature in Bangladesh, (**B**) monthly rainfall in Bangladesh, (**C**) monthly temperature in Australia, (**D**) monthly rainfall in Australia, and (**E**) NINO 3.4 index values decomposed in smooth trend and seasonal effect. Wavelet power values increased from blue to red, and black contour lines indicate a 5% significance level.

There were strong and significant seasonal patterns of 12 months for temperature and rainfall, both in Bangladesh and Australia, as shown by the ACF and wavelet analysis (Figure 2A–D). Significant patterns over 24 and 36 months were observed for NINO 3.4 index (Figure 2E). Moreover, an increasing trend in the global land surface temperature anomalies was observed from 1990 to 2020 (Figure S1).

**Figure 3:**
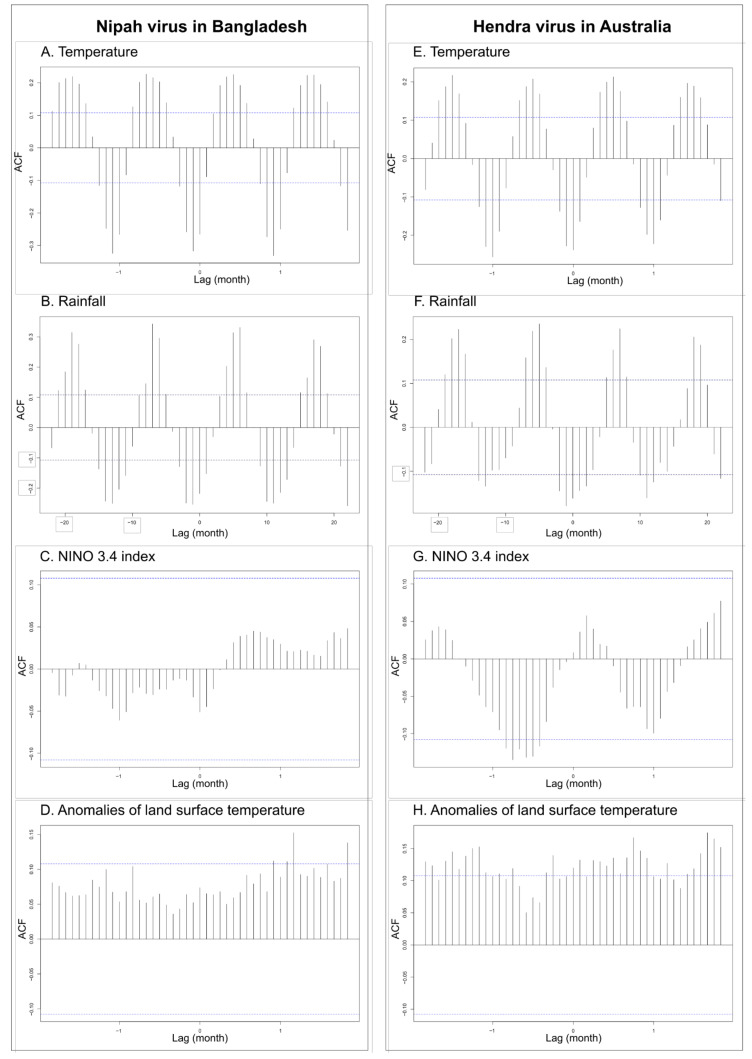
Temporal correlation from January 1993 to June 2020 (330 months) with significant auto-correlation values in dashed lines between spillover events of the Nipah virus in Bangladesh and the Hendra virus in Australia, as well as the monthly temperature (**A**,**E**), the monthly rainfall (**B**,**F**), the NINO 3.4 index (**C**,**G**), and anomalies of the land surface temperature (**D**,**H**).

Cross-correlation analysis among pairs of temporal series of spillover events of NiV and HeV revealed several significant correlations with climate variables (Figure 3; Table 4). Significant correlations were observed for recurring spillover events of NiV with monthly rainfall (lag of 1 month), monthly temperature (lag of 1 month), and land surface temperature anomalies (lag of 10 months) (Figure 3A,B,D; Table 4). No significant correlation was observed for recurring spillover events of NiV with NINO 3.4 index values, although the best correlation was observed for no lag (Figure 3C; Table 4). Significant correlations were observed for recurring spillover events of HeV with NINO 3.4 index values (lag of 7 months), monthly rainfall (lag of 1 month), monthly temperature (no lag), and land surface temperature anomalies (lag of 3 months) (Figure 3E–H; Table 4). Cross-correlation analysis among pairs of temporal series of monthly rainfall, monthly temperature, and land surface temperature anomalies revealed few significant correlations with NINO 3.4 index values (Table 4). There was a significant correlation between land surface temperature anomalies and NINO 3.4 index values (lag of 3 months) and a significant correlation between monthly rainfall in Australia and NINO 3.4 index values (no lag) (Table 4). Non-significant correlations were observed for the monthly temperature in Australia (lag of 7 months), as well as for the monthly temperature and monthly rainfall in Bangladesh (with lags of 10 and 11 months, respectively) (Table 4). 

### 3.2. Logistic Regression Analyses

The above results were used to build two initial logistic regression models based on the lag values obtained by the time-series cross-correlation analysis (see Figure 3 and Table 4). The selected model of the recurring spillover events of HeV in Australia show the significant effects of temperature (with no lag), the global land surface temperature anomalies (with a lag of 3 months), and NINO 3.4 index values (with a lag of 7 months). Rainfall was retained as a variable in the best explanatory model for HeV but had no significant effect(Table 5). The selected model of the recurrent spillover events of NiV in Bangladesh show the only significant effect of rainfall (with a lag of one month), but no effects of global land surface temperature anomalies, the NINO 3.4 index, and the mean temperature (Table 5).

### 3.3. Structural Equation Modelling

**Figure 4:**
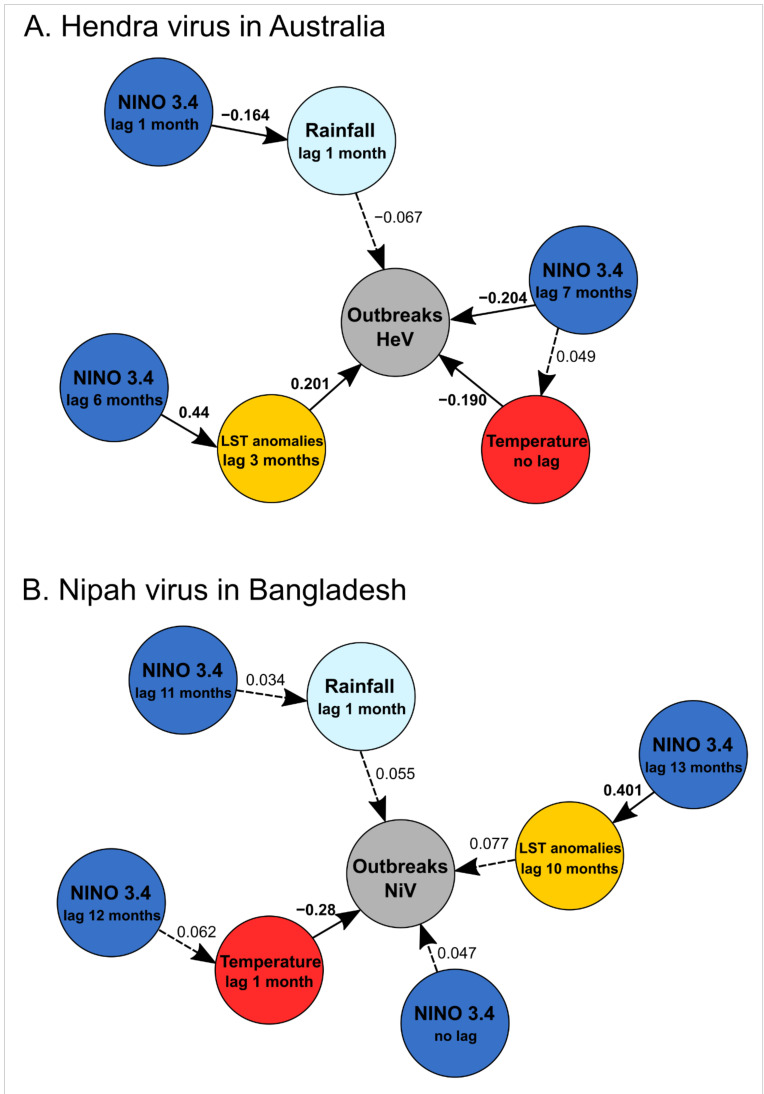
Results of structural equation modelling of (**A**) spillover events of the Hendra virus in Australia and (**B**) spillover events of the Nipah virus in Bangladesh on temporal trends from January 1993 to June 2020 (330 months) based on results obtained from temporal series analyses (Figure 2) and logistic regression analyses with lags (Table 4). Significant partial correlations are presented in continuous lines and non-significant partial correlations are presented in dashed lines with values of standardized estimates.

SEM confirmed the above results for HeV. Significant correlations were observed between the series of recurring HeV spillovers and the mean monthly temperatures in Australia (with no lag, *p* = 0.003), the anomalies in the land surface temperature (with a lag of 3 months, *p* < 0.001), and NINO 3.4 index values (with a lag of 7 months, *p* < 0.001) (Figure 4A; Table 6). The mean monthly rainfall (with a lag of 1 month) and anomalies of the land surface temperature (with a lag of 3 months) were also significantly correlated with NINO 3.4 index values (*p* = 0.003 and *p* < 0.001, respectively) (Figure 4A; Table 6), with respective lags taking into account their estimated values given in Table 4. 

We included the same variables in SEM for NiV and HeV using the lag with the best correlation, although some were non-significant (Table 4). SEM shows that the series of recurring NiV spillovers was only correlated with the mean monthly temperature in Bangladesh (with a lag of one month, *p* < 0.001) (Table 6; Figure 4B), while the monthly rainfall had no significant effect, contrary to what was suggested by the GLM (Table 5). The land surface temperature anomalies were evidently significantly correlated with NINO 3.4 index values (*p* < 0.001), with lag taking into account values given in Table 4. 

### 3.4. Event Coincidence Analysis

**Figure 5:**
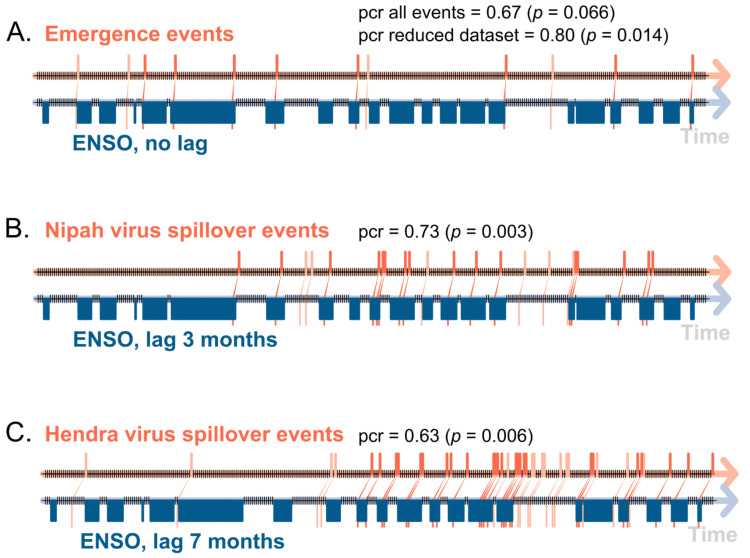
Event coincidence analyses of the association between (**A**) an emergence event of bat-borne virus in the Asia–Pacific region and the Arabian Peninsula, (**B**) a spillover event of the Nipah virus in Bangladesh, and (**C**) a spillover event of the Hendra virus in Australia with an ENSO event (El Niño or La Niña phases) with values of precursor coincidence rate (pcr) and its associated probability and lag value between paired events (lags values used for Nipah virus and Hendra virus analyses correspond to the results of cross-temporal series correlations, shown in Figure 3 and Table 4).

Using the locations and dates of the emergence, as well as the series of spillover events of bat-borne viruses (Table 1, Table 2 and Table 3) and the corresponding ENSO phases (warm, neutral, cool) (Figure 1B; Table 1), we tested the hypothesis that the outbreaks of bat-borne viral diseases were directly preceded (no lag) by an ENSO-driven El Niño/La Niña climate event. The results of the ECA show a random association between an emergence event of bat-borne viral disease (Table 1) following an El Niño/La Niña event (*n* = 12) given by the non-significant value of the precursor coincidence rate (0.67, *p*= 0.066) and the non-significant value of the trigger coincidence rate (0.05, *p* = 0.28) (Figure 5A). However, a non-random association was observed with a significant value of precursor coincidence rate (0.80, *p*= 0.014) when the Australian bat lyssavirus (1996) and the Nipah virus in the Philippines (2014) were removed, as other factors may have impacted these emergence events, as explained above. 

Even if there were no significant associations between NINO 3.4 index values and the NiV outbreak events using time-series analyses or SEM (see above), we explored a possible association using event coincidence analysis. We observed a random statistical relationship between an outbreak event of NiV in Bangladesh (Table 2) and events of El Niño/La Niña (*n* = 22) with no lag (best correlation observed in our cross-correlation analysis, as shown in Table 4). However, a non-random statistical and highly significant relationship between an outbreak event of NiV in Bangladesh and events of El Niño/La Niña using a lag of 3 months was observed, with a significant precursor coincidence rate (0.73, *p*= 0.003) and the non-significant value of a trigger coincidence rate (0.10, *p* = 0.09), suggesting a global lag effect of an ENSO event, whatever its phase (El Niño or La Niña) (Figure 5B). There was a non-random statistical relationship observed between an outbreak event of HeV in Australia (n = 40) following an event of El Niño/La Niña (a significant precursor coincidence rate = 0.63, *p*= 0.006; a trigger coincidence rate = 0.14, *p* = 0.15) (Figure 5C)), with a lag of 7 months estimated by cross-correlation time-series analysis (Figure 3G; Table 4). 

## 4. Discussion

Numerous studies have investigated the origins and drivers of emergence of bat-borne viruses [44,45,46,47,48], with several pinpointing the importance of climate factors and their variability [49,50]. Abnormal rainfall, temperature, and vegetation development associated with ENSO climatic anomalies are known to create appropriate ecological conditions for pathogen emergence, transmission, and propagation [3]. Here, our main objective was to further investigate the potential statistical correlation between emergence and spillover events of several bat-borne viruses and climate factors, such as rainfall, temperature, global surface temperature anomalies, and ENSO events, using a single analytical framework. We used diverse methodologies, such as time-series analyses, logistic regression, SEM, and ECA, to better depict the complex relationships between these climate factors, variability, and anomalies, as well as the emergence and spillover events of bat-borne viruses in the Asia-Pacific region and the Arabian Peninsula. 

While it has already been suggested that the emergences of HeV in Australia in 1994 and NiV in Malaysia in 1998 were associated with El Niño events [18,19,51], the long-term surveillance data of NiV in Bangladesh and HeV in Australia provided a better assessment of the influence of climate variability on the recurring spillovers of these bat-borne viruses. Our findings revealed that the spillover patterns of these two closely related paramyxoviruses, both belonging to the genus henipavirus and with closely related flying fox hosts (*Pteropus* spp.), are differently impacted by climate variability and with different time lags, according to our time-series cross-correlation analysis.

NiV outbreaks occurred almost annually in Bangladesh since 2001, following a seasonal pattern with most outbreaks occurring during the winter months [52]. Even if some livestock and domestic animals were found infected by NiV in Bangladesh [53], contact with sick livestock or domestic animals is not considered an important risk factor of spillover infections in Bangladesh [54]. The seasonal timing and spatial distribution of outbreaks coincide with patterns of raw date palm sap production and consumption [52], suggesting that human behavior and the consumption of date palm sap contaminated by *Pteropus* bats play an important role in these spillovers [54]. However, the fact that the number of NiV spillover events varies greatly from year to year suggests that additional factors influencing bat ecology and movement must be at play [55,56,57]. This was confirmed by a serological survey of *Pteropus medius* bats in Bangladesh which indicated that NiV viral shedding by bats can happen at any time of year and that viral dynamics are cyclical, but not annual or seasonal [58].

So far, a single climatic factor, winter temperature, was shown to be linked to NiV spillover, with colder winter temperatures being associated with more spillovers [56]. Our study confirms the influence of temperature, but our logistic regression models also suggest a correlation between NiV spillover and monthly rainfall, with a short time lag of one month, with lower rainfall being associated with more spillover events. Our cross-correlation analysis, logistic regression models, and SEM did not detect any significant direct correlation between the NINO 3.4 index and NiV spillover events. However, our ECA findings suggest that NiV spillovers in Bangladesh were significantly associated with ENSO events, either El Niño or La Niña phases, with a time lag of three months. This suggests that climate anomalies related to both warm and cool ENSO events may be linked to increased risk of NiV spillover from bats in Bangladesh, which may be explained by the fact that El Niño and La Niña events are characterized by similar rainfall and temperature anomalies in large regions of Bangladesh [59]. ENSO events are associated with the incidence of other diseases in Bangladesh, such as dengue and cholera [60,61]. Climate change will lead to warmer winter temperatures in Bangladesh over the next few decades [62], which may reduce the number of NiV spillover events in the country, as the negative correlation between temperature and NiV outbreaks was clearly demonstrated in this study and others [56]. However, droughts are expected to significantly increase in some regions of Bangladesh under global warming [62,63]. This could negatively impact bat food resources, induce increased bat movement, and potentially lead to more NiV spillover events, as shown by the negative correlation between rainfall and NiV spillover events observed in this study. 

Our study revealed a stronger correlation between climate variability and the spillover pattern of HeV in eastern Australia than NiV in Bangladesh. HeV prevalence in flying foxes in Australia has shown multi-year inter-epidemic periods, suggesting that viral dynamics are not annual, but the ecological drivers and the climate influence behind this pattern remain unclear [64,65,66]. Numerous factors including food shortage, low concentration of nectar-based resources, extreme temperatures, dry conditions, phenology of eucalypt forests, physiological stress, flying fox foraging behavior, and use of wintering roosts in urban and agricultural areas were all suggested to be associated with increased HeV shedding in Australian flying foxes [18,66,67]. Our logistic regression models and SEM show that seasonal climate factors (monthly temperature), but also multi-annual climate variability (ENSO 3.4 index) and long-trend climate anomalies (land surface temperature anomalies), significantly influence the complex pattern of HeV spillover events in Australia. Our ECA also confirmed the hypothesis that HeV outbreaks were preceded by an ENSO-driven El Niño/La Niña climate event with a time lag of seven months. Interestingly, our results show no time lag between the mean monthly temperature recorded in Australia and the spillovers of HeV, suggesting a direct influence of climate seasonality. McMichael et al. [68] hypothesized that this correlation between lower winter temperature and increased HeV shedding in flying foxes could be mediated by the physiological cost of thermoregulation. The temporal lags observed between the ENSO 3.4 index (7 months) or the anomalies of the land surface temperature (3 months) suggest an indirect effect of the climate variability through ecological cascades that may affect food availability, bat migration patterns, and physiological stresses. A previous study [18] showed that the significant impact of ENSO on the flowering phenology of eucalypt, and consequently on bat foraging activities, was characterized by a time lag (3–8 months) similar to the one we observed between ENSO and HeV spillover events in this study (7 months). Lower eucalypt flowering and bat foraging activities induced by an El Niño event may lead to increased HeV prevalence a few months later [18].

Stress induced by climate variability can have a profound effect on disease dynamics in wild animal populations, mostly in relation to immune changes [69] or behavioral changes, such as climate-driven temporary migrations [18]. Bats undergo seasonal physiological changes, including immunological functions, which affect viral shedding [69]. Flying fox immunocompetence is challenged during food shortages driven by climatic anomalies, and HeV (sero) prevalence in Australian pteropid bats increased when their body condition decreased [67,70]. Immunological stress caused by physiological and behavioral changes during the breeding season has been suggested as a contributing factor in HeV shedding in some studies [67], but has been found to have no effect in others [70]. 

Beyond NiV and HeV, our findings suggest that the emergence of most viral diseases of bat origin was likely driven by ENSO climatic anomalies, as 9 out of 12 bat-borne viruses emerged in the Asia–Pacific region and the Arabian Peninsula after an ENSO event over the last three decades (Table 1; Figure 1). Removing the emergence of the Australian bat lyssavirus and NiV in the Philippines, given that other factors may have impacted these emergence events, gave a high prior probability for the emergence of bat virus after an event of El Niño/La Niña in our ECA (Figure 5). The recent emergence of SARS-CoV-2, responsible of the coronavirus disease COVID-19 in China in late 2019, also followed an important El Niño event, which had particularly affected China [71]. 

The emergence of Nipah in the Philippines in March 2014 was not linked to a warm or cool ENSO phase, but did occur following major volcanic activity at Mt Mayon that started one year prior to the emergence [43]. A study conducted after the more recent eruption of Mt. Mayon in 2018 showed large vegetation and environmental impacts of this eruption, which have affected the whole archipelago up to the northern part of Borneo [72]. Studies have also stressed the likely impacts of volcanic activities on disease outbreaks [73]. 

Retrospectively, our findings question the absence of emergence reports during major El Niño-La Niña events before 1994. This may be related to an important limitation of our study and the fact that several past spillover events of bat-borne viruses likely remained undetected. The successful detection of spillover events requires an efficient surveillance system adapted to wildlife, or bats in this case, and many countries are still lacking such a wildlife and human health surveillance system [74]. It is also important to note that climate is not the only factor influencing the emergence of bat-borne viruses. Several additional key drivers that promote cross-species transmission and emergence of zoonotic pathogens have been identified in Asia and include rapidly urbanizing populations, widespread wildlife trade and wildlife consumption, intensive livestock production, deforestation, habitat fragmentation, land-use change, and biodiversity loss [75,76]. Therefore, the absence of bat-borne virus emergence report before 1994 may also be explained by increased contact between bat and human/livestock populations over the last three decades due to land-use change, deforestation, intensification of farming practice, and the expansion of the distribution range of certain bat species linked to climate changes [49,75,76]. 

Climate modelling strongly suggests an intensification of extreme El Niño events in the future [77,78], which will potentially increase the occurrence and outbreaks of infectious diseases and the emergence of bat-borne viral diseases. Climate change will also continue to shift the global distribution of bats and drive changes in bat richness which will increase the risk of bat-borne coronaviruse emergence in the near future [49]. This study and its findings also stress the necessity of improving our knowledge of bat ecology. Close monitoring of bat populations will improve our understanding of viral spillover mechanisms, as demonstrated for HeV in eastern Australia and NiV in Bangladesh, and will contribute to better prediction and prevention strategies.

## Figures and Tables

**Table 1:** Emergence of bat-borne viruses in the Asia–Pacific region and the Arabian Peninsula in relation to El Niño Southern Oscillation (ENSO)-driven climate anomalies (data on ENSO were retrieved from NOAA).

Emergence	Viral Family	Natural Reservoir	Intermediate host	Date, Location	ENSO Phase	References
Hendra virus	Paramyxoviridae	Pteropodid bats	Horse	Aug 1994, Australia	Warm Phase/El Niño	Giles et al., 2018 [18]
Australian bat lyssavirus	Rhabdoviridae	Pteropodid bats	None	Oct 1996, Australia	Neutral Phase	Field et al., 1999 [26]
Menangle virus	Paramyxoviridae	Pteropodid bats	Pig	Jun 1997, Australia	Warm Phase/El Niño	Chant et al., 1998 [27]
Nipah virus	Paramyxoviridae	Pteropodid bats	Pig	Sep 1998, Malaysia	Cool Phase/La Niña	Ang et al., 2018 [25]
Nipah virus	Paramyxoviridae	Pteropodid bats	None	Jan 2001, India	Cool Phase/La Niña	Ang et al., 2018 [25]
SARS-CoV-1	Coronaviridae	Rhinolophid bats	Small carnivores	Nov 2002, China	Warm Phase/El Niño	Ge et al., 2013 [28]
Melaka virus	Reoviridae	Pteropodid bats	None	Mar 2006, Malaysia	Cool Phase/La Niña	Chua et al., 2008 [29]
Kampar virus	Reoviridae	Pteropodid bats	None	Aug 2006, Malaysia	Neutral Phase	Chua et al., 2008 [29]
MERS-CoV	Coronaviridae	Vespertilionid bats	Camel	Apr 2012, Middle East	Cool Phase/La Niña	Zaki et al., 2012 [30]
Nipah virus	Paramyxoviridae	Pteropodid bats	Horse	Mar 2014, The Philippines	Neutral phase	Ching et al., 2015 [31]
SADS-CoV	Coronaviridae	Rhinolophid bats	Pig (no human cases)	Oct 2016, China	Cool Phase/La Nina	Gong et al., 2017 [32]
SARS-CoV-2	Coronaviridae	Rhinolophid bats	?	December 2019, China	Warm Phase/El Niño	Zhu et al., 2020 [33]

**Table 2:** Recurring spillover events of the Nipah virus after its first emergence in South Asia (India and Bangladesh) in relation to El Niño Southern Oscillation (ENSO)-driven climate anomalies (data on ENSO were retrieved from NOAA). Data on outbreaks were retrieved from Rahman and Chakraborty (2012) [23], Ang et al. (2018) [25], and Rahman et al. (2021) [24].

Country	Date (Month/Year)	ENSO Phase
Bangladesh	April 2001	Neutral Phase
Bangladesh	January 2003	Warm Phase/El Niño
Bangladesh	January 2004	Neutral Phase
Bangladesh	April 2004	Neutral Phase
Bangladesh	January 2005	Warm Phase/El Niño
Bangladesh	January 2007	Warm Phase/El Niño
Bangladesh	March 2007	Neutral Phase
Bangladesh	April 2007	Neutral Phase
India	April 2007	Neutral Phase
Bangladesh	February 2008	Cool Phase/La Niña
Bangladesh	April 2008	Cool Phase/La Niña
Bangladesh	January 2009	Cool Phase/La Niña
Bangladesh	February 2010	Warm Phase/El Niño
Bangladesh	January 2011	Cool Phase/La Niña
Bangladesh	January 2012	Cool Phase/La Niña
Bangladesh	January 2013	Neutral Phase
Bangladesh	January 2014	Warm Phase/El Niño
Bangladesh	January 2015	Warm Phase/El Niño
Bangladesh	February 2015	Neutral Phase
Bangladesh	March 2015	Warm Phase/El Niño
Bangladesh	February 2017	Neutral Phase
Bangladesh	February 2018	Cool Phase/La Niña
Bangladesh	April 2018	Cool Phase/La Niña
India	May 2018	Neutral Phase
India	June 2019	Neutral Phase

**Table 3:** Recurring spillover events of the Hendra virus after its first emergence in Australia in relation to El Niño Southern Oscillation (ENSO)-driven climate anomalies (data on ENSO were retrieved from NOAA). Data on outbreaks were retrieved from the Queensland Government database (https://www.business.qld.gov.au/industries/service-industries-professionals/service-industries/veterinary-surgeons/guidelines-hendra/incident-summary, accessed on 17 May 2022).

Country	Date (Month/Year)	ENSO Phase
Australia	September 1994	Warm Phase/El Niño
Australia	January 1999	Cool Phase/La Niña
Australia	October 2004	Warm Phase/El Niño
Australia	December 2004	Warm Phase/El Niño
Australia	June 2006	Neutral Phase
Australia	October 2006	Warm Phase/El Niño
Australia	June 2007	Neutral Phase
Australia	July 2007	Cool Phase/La Niña
Australia	June 2008	Cool Phase/La Niña
Australia	July 2008,	Neutral Phase
Australia	July 2009	Neutral Phase
Australia	September 2009	Warm Phase/El Niño
Australia	May 2010	Neutral Phase
Australia	June 2011 (4 events)	Cool Phase/La Niña
Australia	July 2011 (8 events)	Neutral phase
Australia	August 2011 (5 events)	Cool Phase/La Niña
Australia	October 2011	Cool Phase/La Niña
Australia	January 2012	Cool Phase/La Niña
Australia	May 2012 (2 events)	Neutral Phase
Australia	June 2012	Neutral Phase
Australia	July 2012 (2 events)	Neutral Phase
Australia	September 2012	Neutral Phase
Australia	October 2012	Neutral Phase
Australia	January 2013	Neutral Phase
Australia	February 2013	Neutral Phase
Australia	June 2013 (2 events)	Neutral Phase
Australia	July 2013 (4 events)	Neutral Phase
Australia	March 2014	Neutral Phase
Australia	June 2014 (2 events)	Neutral Phase
Australia	July 2014	Neutral Phase
Australia	June 2015	Warm Phase/El Niño
Australia	July 2015	Warm Phase/El Niño
Australia	September 2015	Warm Phase/El Niño
Australia	December 2016	Cool Phase/La Niña
Australia	May 2017	Neutral Phase
Australia	July 2017	Neutral Phase
Australia	August 2017 (2 events)	Neutral Phase
Australia	September 2018	Neutral Phase
Australia	June 2019	Warm Phase/El Niño
Australia	June 2020	Neutral Phase

**Table 4:** Results of temporal cross-association between spillover events of the Hendra virus (HeV), the Nipah virus (NiV), NINO 3.4 index, the average monthly temperature in Australia and Bangladesh, the average monthly rainfall in Australia and Bangladesh, and land surface temperature anomalies (monthly lag values were obtained from time-series cross-correlation analysis). Significant correlations are highlighted in bold.

First Time-Series	Second Time-Series	Lag	Correlation (*p* Value)
Spillover events of HeV (Australia)	NINO 3.4 index	7 months	**0.13 (0.018)**
	Rainfall (Australia)	1 month	**0.18 (0.002)**
	Temperature (Australia)	0 month	**0.24 (< 0.001)**
	Land surface temperature anomalies	3 months	**0.14 (0.012)**
Spillover events of NiV (Bangladesh)	NINO 3.4 index Rainfall (Bangladesh)	0 month 1 month	0.05 (0.35 **0.22 (0.008)**
	Temperature (Bangladesh)	1 month	**0.32 (0.008)**
	Land surface temperature anomalies	10 months	**0.10 (0.047)**
NINO 3.4 index	Rainfall (Australia)	0 month	**0.16 (0.007)**
	Temperature (Australia)	7 months	0.08 (0.35)
	Rainfall (Bangladesh)	10 months	0.03 (0.58)
	Temperature (Bangladesh)	11 months	0.06 (0.27)
	Land surface temperature anomalies	3 months	**0.40 (<0.0001)**

**Table 5:** Results of the logistic regression modelling with lags to explore the temporal association between spillover events of the Hendra virus (HeV) and the Nipah virus (NiV) from January 1993 to June 2020. The initial models included the following variables: the NINO 3.4 index, the average monthly temperature in Australia/Bangladesh, the average monthly rainfall in Australia/Bangladesh, and the land surface temperature anomalies with lag values obtained from time-series cross-correlation analysis (Table 4). The best explanatory models were selected using a backward procedure with AIC criterion. Significant *p*-values are highlighted in bold.

Response Variable	Predictor Variable	Estimate (Std Err)	Odds Ratio (2.5–97.5 %)	*p*	R2 (Global)
HeV spillover events (Australia)	NINO 3.4 index (lag = 7 months)	−0.72 (0.23)	0.49 (0.30–0.75)	**0.002**	
	Rainfall (lag = 1 month)	−0.01 (0.01)	0.99 (0.96–1.00	0.16	
	Temperature (lag = 0 month)	−0.11 (0.04)	0.90 (0.82–0.98)	**0.013**	
	Land surface temperature anomalies (lag = 3 months)	3.43 (1.03)	30.96 (4.32–254.30)	**0.001**	0.21
NiV spillover events (Bangladesh)	Rainfall (lag = 1 month)	−0.03 (0.01)	0.97 (0.95–0.99)	**0.008**	0.30

**Table 6:** Results of the structural equation modelling (SEM) to explore the temporal associations between spillover events of the Hendra virus (HeV) or the Nipah virus (NiV), the monthly temperature, the monthly rainfall, the NINO 3.4 index, and land surface temperature (LST) anomalies from January 1991 to June 2020. Lag values were obtained from the time-series analyses and the logistic regression analyses (Table 4). Significant *p*-values are highlighted in bold.

Model	Response Variable	Predictor Variable	Estimate (Std Err), df	Standardized Estimate	*p*	R2 (Individual)
HeV	Spillover events of HeV	‘NINO 3.4′ (lag = 7 months)	−0.077 (0.022), 313	−0.204	**<0.001**	
		Rainfall (lag = 1 month)	−0.001 (0.001), 313	−0.067	0.28	
		Temperature (lag = 0 month)	−0.078 (0.022), 313	−0.190	**0.003**	
		LST anom (lag = 3 months)	0.3123 (0.095), 313	0.201	**<0.001**	0.11
	Rainfall (lag = 1 month)	‘NINO 3.4′ (lag = 1 month)	−5.223 (1.7670), 316	−0.1638	**0.003**	0.027
	Temperature	‘NINO 3.4′ (lag = 7 months)	0.271 (0.313), 316	0.049	0.389	0.002
	LST anomalies (lag = 3 months)	‘NINO 3.4′(lag = 6 months)	0.101 (0.012), 316	0.434	**<0.001**	0.19
NiV	Spillover events of NiV	‘NINO 3.4′ (lag = 0 month)	−0.014 (0.015), 312	−0.887	0.38	
		Rainfall (lag = 1 month)	−0.0001 (0.0001), 312	−0.718	0.47	
		Temperature (lag = 1 month)	−0.020 (0.005), 312	−3.689	**0.003**	
		LST anom (lag = 10 month)	0.098 (0.067), 312	1.443	0.15	0.12
	Rainfall (lag = 1 month)	NINO 3.4′ (lag = 11 months)	6.730 (11.178), 315	0.602	0.55	0.001
	Temperature (lag = 1 month)	NINO 3.4′ (lag = 12 months)	0.254 (0.231), 315	1.100	0.272	0.004
	LST anomalies (lag = 10 months)	‘NINO 3.4′ (lag = 13 months)	0.092 (0.012), 315	7.774	**<0.001**	0.16

## Data Availability

Data and codes used in the used in the study are available as Supplementary Materials (Table S1, R code S1, R code S2).

## Supplementary Materials

The following supporting information can be downloaded at: https://www.mdpi.com/article/10.3390/v14051100/s1. Figure S1: Global land surface temperature anomalies 1993–2020. Table S1: Emergence and spillover data used in the study. R code S1: R code used to conduct the analyses described in the study. R code S2: R code used to obtain Figure 1B.
