# Supplementary figures and images for: Climate Anomalies and Spillover of Bat-Borne Viral Diseases in the Asia–Pacific Region and the Arabian Peninsula
Source: Viruses. 2022 May 20;14(5):1100. doi: 10.3390/v14051100 (PMC9145311; doi:10.3390/v14051100)

# Land Surface Temperature Anomaly

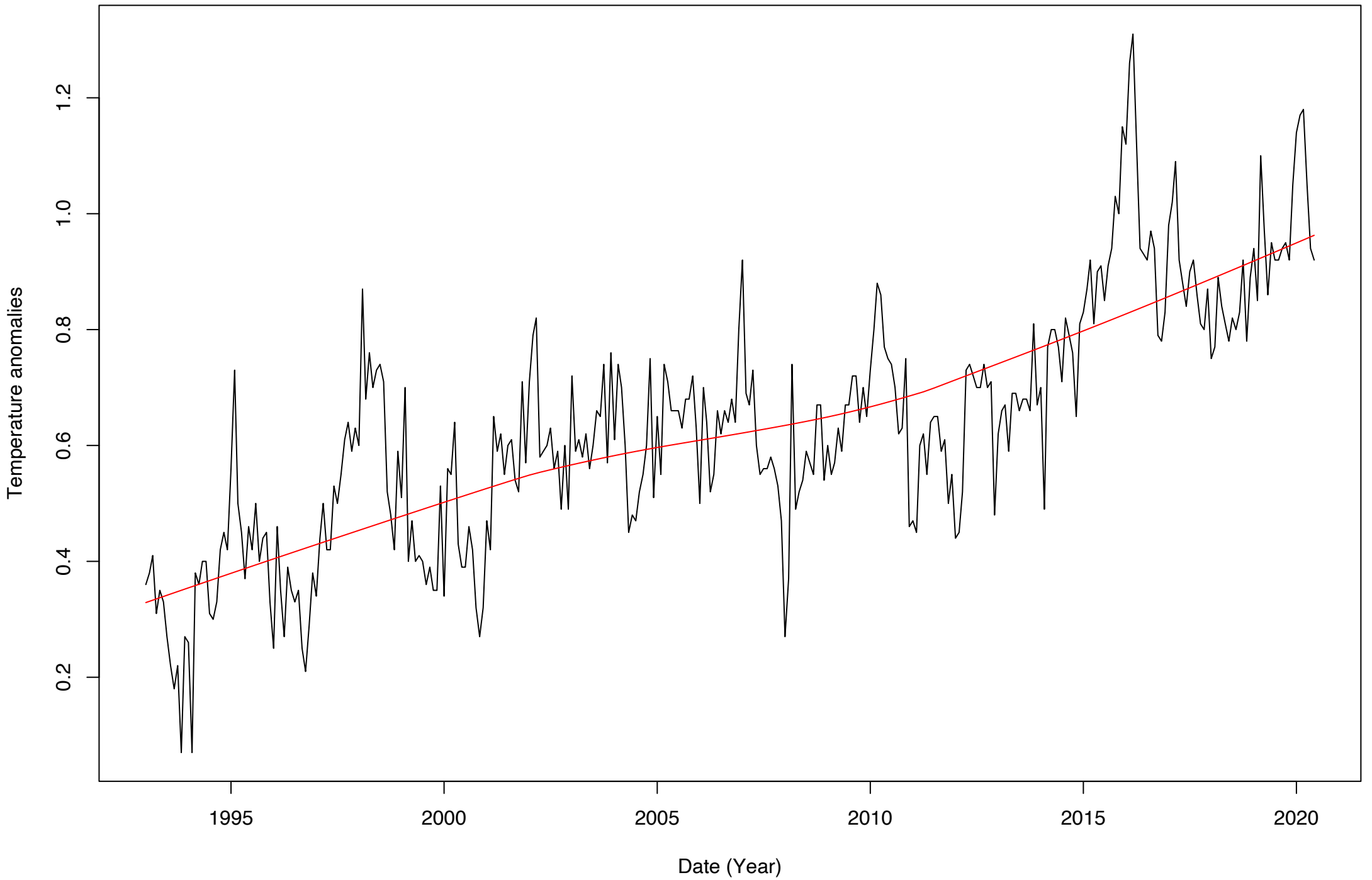

Supplement: Supplementary file 1 [file viruses-14-01100-s001.zip › FigS1 Land Surface anomalies.pdf]
